# High-dose rate brachytherapy (HDRB) for primary or recurrent cancer in the vagina

**DOI:** 10.1186/1748-717X-3-7

**Published:** 2008-02-13

**Authors:** Sushil Beriwal, Dwight E Heron, Robert Mogus, Robert P Edwards, Joseph L Kelley, Paniti Sukumvanich

**Affiliations:** 1 Department of Radiation Oncology, University of Pittsburgh Cancer Institute, Pittsburgh, PA, USA; 2 Division of Gynecologic Oncology, Department of Obstetrics, Gynecology and Reproductive Sciences, Magee-Womens Hospital, University of Pittsburgh Medical Center, Pittsburgh, PA, USA

## Abstract

**Purpose:**

The purpose of this study was to evaluate the efficacy of HDR brachytherapy for primary or recurrent vaginal cancer.

**Methods:**

Between the years 2000 to 2006, 18 patients with primary or recurrent vaginal cancer were treated with brachytherapy (HDRB). Six patients had primary vaginal cancer (stage II to IVA) while 12 were treated for isolated vaginal recurrence (primary cervix = 4, vulva = 1 and endometrium = 7). Five patients had previous pelvic radiation therapy. All except one patient received external beam radiation therapy to a median dose of 45 Gy (range 31.2–55.8 Gy). The HDRB was intracavitary using a vaginal cylinder in 5 patients and interstitial using a modified Syed-Nesblett template in 13 patients. The dose of interstitial brachytherapy was 18.75 Gy in 5 fractions delivered twice daily. The median follow-up was 18 months (range 6–66 months).

**Results:**

Complete response (CR) was achieved in all but one patient (94%). Of these 17 patients achieving a CR, 1 had local recurrence and 3 had systemic recurrence at a median time of 6 months (range 6–22 months). The 2-year actuarial local control and cause-specific survival for the entire group were 88% and 82.5%, respectively. In subset analysis, the crude local control was 100% for primary vaginal cancer, 100% for the group with recurrence without any prior radiation and 67% for group with recurrence and prior radiation therapy. Two patients had late grade 3 or higher morbidity (rectovaginal fistula in one patient and chronic vaginal ulcer resulting in bleeding in one patient). Both these patients had prior radiation therapy.

**Conclusion:**

Our small series suggests that HDRB is efficacious for primary or recurrent vaginal cancer. Patients treated with primary disease and those with recurrent disease without prior irradiation have the greatest benefit from HDRB in this setting. The salvage rate for patients with prior radiation therapy is lower with a higher risk of significant complications. Additional patients and follow-up are ongoing to determine the long-term efficacy of this approach.

## Background

Primary or recurrent vaginal carcinoma is an uncommon tumor [1]. The initial tumor volume, tumor extent within the vagina, histologic type and grade, lymphatic involvement and previous treatment are all important determinant for overall outcome. Although surgical resection of the tumor is occasionally possible, radiation therapy is currently the standard treatment for this disease [2-6]. The radiation therapy regimen includes external beam radiation therapy (EBRT), brachytherapy, or a combination thereof.

Brachytherapy has been shown to be an important component of treatment in these patients. Treatment selection can be adapted to account for stage and location of the tumor. It can be done with either intracavitary or interstitial approach. The majority of published studies with interstitial brachytherapy have reported data using low-dose-rate brachytherapy (LDRB) [2-9]. There have been only few series using high-dose-rate brachytherapy (HDRB) for vaginal tumors [10-12].

The purpose of this study was to evaluate the efficacy and toxicities of HDRB for primary or recurrent vaginal cancer.

## Methods

Between January 2000 and December 2006, 18 patients with primary or recurrent vaginal cancer were treated with HDRB. The median age was 69 yrs (range 43–88 yrs). Six patients had primary vaginal cancer (stage II – 4 patients, stage III – 1 patient and stage IV A – 1 patient). Twelve patients were treated for isolated vaginal recurrence (primary cervix = 4, vulva = 1 and endometrium = 7). The stage and grade (G) of endometrial cancer were IB G1-1 patient, IB G2-2 patients, IC G3-2 patient, IIA G 3 – 1 patient and IIB G2 – 1 patient. The median time to recurrence for all patients was 12 months (range 3 months – 18 years). Six of these patients had prior pelvic radiation therapy. The type of previous radiation was EBRT alone 1 patient, EBRT plus brachytherapy 3 patients and brachytherapy alone 2 patients. The median time since previous radiation to recurrence was 4 years (6 months – 18 years). The recurrence was marginal (within 2 cm of previous field) in 2 patients and within the previous field in 4 patients. The sites of disease were proximal vagina 9 patients, distal vagina 6 patients and diffuse disease in 3 patients.

All except one patient received a combination EBRT and HDRB. The median dose of EBRT was 45 Gy (range 31.2–55.8 Gy) at 1.8 to 2 Gy per fraction. The technique of EBRT was 3D conformal in 9 patients and IMRT in 8 patients. Five out of six patents with vaginal cancer also had concurrent weekly cisplatinum at 40 mg/m2 along with EBRT.

**Figure 1:**
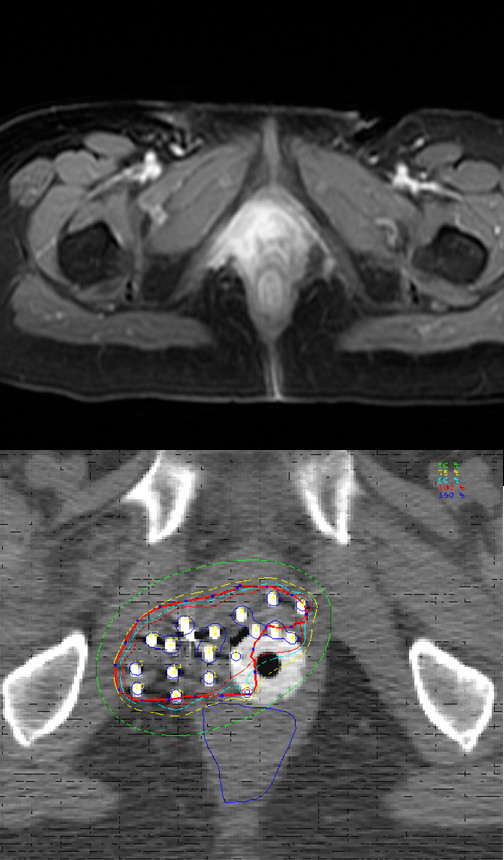
**Pre-RT MRI image (T1 with contrast) showing large vaginal cancer with extension to pelvic side wall and peri-urethral region**. Interstitial implantation showing coverage of clinical tumor volume (red) with isodose lines (100%-red, 90%-blue, 75%-yellow, and 50% – green).

The HDRB was intracavitary brachytherapy for superficially invasive tumors (less than 5 mm of invasion), while interstitial brachytherapy was used for more deeply invasive tumors greater than 5 mm. Five patients had intracavitary brachytherapy utilizing Delclos Vaginal Applicators system. Two of these patients had shielded vaginal applicators to protect the uninvolved vaginal mucosa. Orthogonal X-ray films were obtained for planning and verification of applicator placement. The median dose for intracavitary brachytherapy was 20 Gy (12 – 20 Gy) in 3–5 fractions prescribed at 0.5 cm from the surface of the applicator. One of these patients had intracavitary brachytherapy only because of previous radiation therapy with EBRT plus HDR brachytherapy. Interstitial brachytherapy using a modified Syed-Nesblett template was performed in 13 patients. We have previously described this technique [13]. In brief, the procedure was performed under general anesthesia intra-operatively and epidural analgesia was used to control post-operative pain during the course of treatment. Laparoscopic guidance was used for tumors involving the vaginal apex to avoid injury to the bladder or adjacent small bowel. CT simulation for interstitial brachytherapy planning was performed and the dose was prescribed to clinical target volume defined based on clinical and imaging findings at the time of implantation(Figure 1). Pretreatment clinical findings and imaging were also taken for reference for defining the target volume. The median number of needles used were 14 (range 7–18). The dose of interstitial brachytherapy was 18.75 Gy in 5 fractions delivered twice daily.

The biologically equivalent dose (BED) in terms of equivalent doses given at 2 Gy per day (EQ2) was calculated using the LQ equation [14]. The α/β ratio was taken to be 10 Gy for tumor effects and 3 Gy for late effects. The median EQ2 for the tumor was 66.48 Gy (31.25 – 75.4 Gy) for the entire group. The median EQ2 for the late effects on vaginal mucosa was 75.71 Gy (55.99 – 105.99 Gy). The cumulative EQ2 for late effects on vaginal mucosa for four patients who had prior overlapping field radiation was 120.7 Gy, 130.31 Gy, 142.98 Gy and 154.54 Gy, respectively

The median follow-up was 18 months (range 6–66 months) for the entire group. Complete response (CR) was achieved in all but one patient (94%). This one patient with previous radiation therapy had recurrent papillary serous endometrial cancer and had partial regression of the tumor. Of the 17 patients achieving a CR, 1 had local recurrence and 3 had systemic recurrence at a median time of 6 months (range 6–22 months). The one local recurrence was in the patient treated with previous adjuvant radiation therapy for endometrioid adenocarcinoma. Two patients have died of disease at 12 and 18 months, respectively. Both patients had recurrent endometrial cancer with papillary serous and carcinosarcoma histology, respectively. Persistence of local disease was noted in one of these patients. The 2-year actuarial local control, cause-specific and overall survival for the entire group was 88%, 82.5% and 78%, respectively. In subset analysis, the crude local control was 6/6 (100%) for primary vaginal cancer, 6/6 (100%) for the group with recurrence without any prior radiation and 4/6 (67%) for group with recurrence and any prior radiation therapy.

No grade 3 or worse early toxicity was observed. Two patients had late grade 3 or 4 morbidity. One patient had rectovaginal fistula 2 years after radiation therapy and other patient had chronic vaginal ulcer with significant narrowing and shortening of vagina (length of residual vagina was about 2 cm). Both these patients had proximal vaginal disease and had prior radiation therapy and their EQ 2 for total doses were 142.98 Gy and 154 Gy, respectively. The cumulative doses were highest in these two patients. No other significant grade 3 or higher morbidity was noted.

## Discussion

Radiotherapy is the main therapeutic modality in the management of primary or recurrent vaginal cancer. Brachytherapy remains integral part of definitive radiation therapy for these patients [2-9]. The preponderance of the published literature on brachytherapy in this setting demonstrates a wide variation on techniques and dose utilized for LDRB. Although HDRB is widely available and used for a variety of gynecologic malignancies, the published data on this technique for vaginal cancer treatment are limited. The HDR planning software offers the ability to optimize the dwell time and position of the radioisotope source (Ir-192) in order to create conformal radiation treatment to a specified target. This improvement in dose uniformity and ratios between tumor and normal tissue is important advantage of HDR brachytherapy. These advantages of HDRB over LDRB have to be weighed against the need to use a larger number of HDR fractions and the inconvenience of multiple implantations.

In a review of the literature regarding salvage treatment options for vaginal recurrence, only two studies have examined the efficacy of HDRB for treatment of vaginal recurrences [11,15]. The study by Petignat, *et al. *on 22 patients with recurrent endometrial cancer reported local tumor control rate and the 5-year disease-specific survival rate of 100% and 96%, respectively [11]. Similarly, in the series by Pai, *et al. *on 20 patients, 10-year local control rate and disease-free survival rates were 74% and 46%, respectively [15]. Our study with shorter follow-up shows comparable local control and cause-specific survival. The major difference between the published series and our data is the technique of HDRB. The majority of the patients in either study were treated with intracavitary brachytherapy with only 2 patients having interstitial implantation. In contrast, in our study 13/18 patients had interstitial brachytherapy. The technique of intracavitary is recommended for non-bulky recurrences (thickness < 5 mm after the completion of EBRT) while interstitial brachytherapy is preferred approach for bulky recurrences [16].

Interstitial brachytherapy has been traditionally performed using LDRB techniques in this setting. Our technique involved one Syed-**Neblett **template implantation procedure delivering 5 fractions twice a day for a total HDRB dose of 18.75 Gy over 48 – 56 hours. Because of lack of published data, the American Brachytherapy Society (ABS) did not make any recommendation on HDRB interstitial brachytherapy dose and fractionation schedule for vaginal recurrences and preferred LDRB as the technique for interstitial brachytherapy [16]. Our treatment regimen was well tolerated with excellent local control and low toxicities in patients with no prior radiation.

Similarly, there are only few published studies on HDRB for primary vaginal cancer. Kusher, *et al. *first reported on 19 patients treated with the combination of intracavitary and interstitial brachytherapy [10]. The median HDRB dose was 23 Gy (LDR equivalent of 29.8 Gy) after median EBRT dose of 40 Gy. The 2-year progression-free survival was 39.3% while the 2-year overall survival was 66.1%. Three patients developed serious and/or late complications including urethral stenosis, painful vaginal necrosis and small bowel obstruction of which two had interstitial brachytherapy. The largest series of HDRB for vaginal cancer is from Vienna reporting on a total of 86 patients of primary vaginal carcinoma treated with HDRB [12]. Early stages of disease (stages 0–II) were treated with intravaginal HDR brachytherapy alone (*n *= 26/86), whereas patients with locally advanced disease (stages II-IV) received HDR brachytherapy combined with external beam therapy (*n *= 55/86). The prescribed dose per fraction varied from 5 Gy to 8 Gy, with a mean dose of 7 Gy. In this large series only 8 patients had interstitial brachytherapy. The 5-year recurrence-free survival were 100%, 77%, 50%, 23%, and 0% for stage 0, I, II, III and IV respectively. Chronic grade 1–4 side effects were observed in ≤ 2% (bladder, rectum) and 1%–6% (vagina). Our series only had 6 patients of primary vaginal cancer and all had interstitial brachytherapy with 2-year local control of 100%. The toxicity profile was favorable with no grade 3 or higher toxicities.

In LDRB literature for vaginal cancer, an inverse relationship between the total dose and rates of local recurrences have been reported by several authors [17,18]. Chyle, *et al. *noted an increasing risk of local recurrences in patients who received <55 Gy when compared with those receiving >55 Gy (53% vs. 17%) [12]. Similarly Fine, *et al. *reported local failures in 25%, 33% and 62% of patients for the administered dose of >75 Gy, 60–75 Gy and <60 Gy respectively [18]. Our median EQ2 of 66.48 Gy is comparable with these doses recommended in LDRB literature. Besides, our calculation of biological equivalence is based on the assumption of complete repair of sub-lethal radiation damage between the two fractions [14]. With a twice-daily (BID) fractionation schedule and time interval of 6 hours between fractionation, the sublethal damage may not be complete thereby causing more injury to both tumor and normal tissues than predicted by BED models.

Notwithstanding, our small series with preliminary results shows that HDRB is efficacious for primary or recurrent vaginal cancer. The fractionation schedule used was well tolerated with a low incidence of acute or later toxicities. Additional patients and follow-up are ongoing to determine the long-term efficacy of this approach. The limitation of our retrospective study is small size with limited follow up and heterogeneous patient population evaluated (inclusion of both primary and recurrent disease). The incidence of primary or recurrent vaginal cancer is low so that it is difficult for a single institution to have a large series. That was the rationale to combine heterogeneous disease together to see the efficacy and toxicities of this approach. As HDR equipment is widely available, there are more institution doing HDR interstitial brachytherapy. We may need to consider multi-institutional pooled analysis similar to the LDR experience [19] to see the impact of this technique for local control and toxicities and to define optimal fractionation schedules.

## Authors' contributions

SB – Took part in design and implementation of study, drafted manuscript, performed statistical analysis. DEH – Lead in drafting manuscript. RM – Helped in data collection. RPE – Participated in study design and patient selection. JLK – Participated in study design and patient selection. PS – Conceived the study, participated with design and coordinated/helped with patient selection. All authors read and approved the final manuscript.
